# Systematic surveillance of patient-reported symptoms of viral respiratory tract infectious Syndromes in diverse populations

**DOI:** 10.1186/s12913-022-08991-3

**Published:** 2022-12-29

**Authors:** Jennifer C. Gander, Ella Chrenka, Lee Cromwell, Anjali R. Truitt, Musu Sesay, Marni Segall, Sandra A. Amouzou, Alexander F. Hudgins, Prasanthi Kodthala, Douglas Roblin, Adrienne N. Deneal, Thomas Whiting, John H. Powers, Brian C. Martinson

**Affiliations:** 1 Center for Research and Evaluation, Kaiser Permanente Georgia, Atlanta, GA USA; 2 HealthPartners Institute, Bloomington, MN USA; 3 Mid-Atlantic Permanente Research Institute, Kaiser Permanente Mid-Atlantic States, Rockville, MD USA; 4 George Washington University School of Medicine, Washington, DC USA

**Keywords:** Patient-reported outcome measures, COVID-19, Influenza-like illness, Patient-centered care

## Abstract

**Background:**

Patient reported outcome measures (PROM) can improve patient care and be crucial for symptom tracking especially during disease outbreaks. FLU-PRO Plus is a validated PROM used to track viral respiratory symptoms. Our study aimed to evaluate the feasibility of using FLU-PRO© Plus, to track symptoms across three healthcare systems.

**Methods:**

The prospective, longitudinal study recruited adults between February-May 2021 from HealthPartners Institute (HP), Kaiser Permanente Georgia (KPGA), and Kaiser Permanente Mid-Atlantic States (KPMAS). Adult members were eligible if they had a positive lab or diagnosis for either COVID-19 or influenza-like illness (ILI) or exhibited 2 + viral respiratory symptoms. Descriptive statistics were calculated to describe the patient characteristics for participants that were eligible for FLU-PRO Plus, successfully contacted, attempted to log in to the FLU-PRO Plus website, and participants who completed FLU-PRO Plus Day 1. Bivariable and multivariable logistic regression using PROC GLIMMIXX investigated the patient characteristics associated with (1) successful contact and (2) FLU-PRO Plus Day 1 completion.

**Results:**

We identified a total of 15,650 eligible participants during the enrollment period: 9,582 from HP, 1,740 from KPGA, and 4,328 from KPMAS. Among the total of 409 eligible adults who attempted to participate in FLU-PRO Plus, 317 completed FLU-PRO Plus Day 1. Among the 317 individuals that completed FLU-PRO Plus Day 1, 205 (67.5%) were diagnosed with COVID-19; 112 adults diagnosed with COVID-19 completed FLU-PRO Plus Day 14. Among adults successfully contacted, adults aged 35–64 (OR = 1.40, 95% CI 1.05, 1.87), females (OR = 1.77, 95% CI 1.38, 2.27), and adults diagnosed with COVID-19 (OR = 1.66, 95% CI 1.27, 2.17) had higher odds of completing FLU-PRO Plus Day 1; Asian adults (OR = 0.38, 95% CI 0.19, 0.76) and Black and African American adults (OR = 0.33, 95% CI 0.19, 0.76) had lower odds compared to White adults.

**Conclusion:**

Our study reports on the feasibility of patients across three integrated healthcare systems utilizing FLU-PRO Plus to monitor their respiratory symptoms. Patient reported outcome measures (PROM) can improve patient care, quality of life, and reduce the strain of limited resources on healthcare systems. Future FLU-PRO Plus studies should develop an implementation strategy to fully integrate FLU-PRO Plus within clinical care and patient management.

**Supplementary Information:**

The online version contains supplementary material available at 10.1186/s12913-022-08991-3.

## Introduction

Patient centered care has become increasingly recognized as an integral component and ethical imperative of modern healthcare [1]. Patient reported outcome measures (PROMs) are patient-reported health outcomes without interpretation from healthcare professionals, enabling patients to report what they are experiencing. PROMs have been shown to be significantly predictive of patient health status and to improve patient care [2, 3]. PROMs have been developed and shown to be valuable for patient care in chronic diseases [4–7], cancer [2, 8–14], orthopedics [15], mental health [16], primary care [17], and infectious diseases [18–21]. PROMs used sequentially over a series of several days for patients undergoing cancer treatment enabled the patients’ clinical team to monitor the patient-reported symptoms, provided the participants continuous access to self-care advice, and lessened the physical symptom distress patients experienced while improving their emotional functioning, compared to the patients randomized to usual care [13].

The increased use of telemedicine, ownership of electronic smart devices and availability of telemedicine have led to an increased development of electronic PROMs [22–25]. Electronic PROMs may be administered within a clinic setting or remotely. PROMs can improve symptom management between visits and be a cost-effective options for a healthcare system by minimizing the frequency of costly diagnostic tests and scans [2, 26–28]. Remote use of electronic PROMs with built-in prompts to encourage completion may reduce recall bias by allowing the patient to report their symptoms or outcomes they are experiencing more accurately and promptly [29–31]. However, despite the growing evidence of PROM benefits to the patients and healthcare systems, PROMs have not been widely implemented across healthcare systems or disease spectrums [32].

Viral respiratory illnesses are common, although the symptoms, symptom intensity, and symptom duration can vary across illnesses [28, 31]. The onset of the novel coronavirus (COVID-19) brought about a variety of patient-reported surveys that individuals could use to report and track their symptoms [33–36]. FLU-PRO© is a validated PROM originally developed to standardize capture of patients’ symptoms associated with influenza, respiratory syncytial virus, rhinovirus, enterovirus, and endemic coronaviruses throughout a 14-day course, or until symptoms subside and participants return to their baseline health [28, 31]. The instrument was developed under the US Food and Drug Administration’s recommendations for evaluating content validity and used a two-stage qualitative methodology, concept elicitation and cognitive interviews [28]. The initial, 32-item, version of FLU-PRO contained six domains (nose, throat, eyes, chest/respiratory, gastrointestinal, and body/systemic) and demonstrated an overall comparative fit index = 0.92 [31]. FLU-PRO’s 32-items were revised in 2020 to incorporate a seventh domain of senses (loss of taste and smell) [36]. This PROM asks participants, “please rate the extent to which you had each symptom during the past 24 hours” with Likert Scale answer choices ranging from, “Not at all”, “A little bit”, “Somewhat”, “Quite a bit”, to “Very much”. Domain-specific questions were asked; for example, questions under the ‘eyes’ domain inquire about watery or painful eyes, and the ‘gastrointestinal’ domain contains items on nausea and stomachache. FLU-PRO Plus also requests individuals to report, overall, how their symptoms are today, how their symptoms are compared to yesterday, and the interference their symptoms have on daily activities [36]. Although FLU-PRO© and FLU-PRO Plus have been developed and validated in several clinical trials and prospective cohorts [28, 31, 36], the feasibility of implementing FLU-PRO Plus within healthcare systems has not been evaluated.

Our multi-site study across three geographically diverse integrated healthcare systems aimed to evaluate the feasibility of routine surveillance of respiratory viral syndromes in clinical practice by tracking the number and proportion of patients completing the FLU-PRO Plus symptom questions over a 14-day period. We described the recruitment process and recruitment rate across each healthcare system and compared the participants who completed Day 1 of FLU-PRO Plus to all eligible individuals and individuals who were successfully contacted to participate in FLU-PRO Plus.

## Methods

### Study design and overview

We conducted a prospective longitudinal study evaluating patient-reported respiratory symptoms. Adult (age ≥ 18 years) patients were eligible for recruitment if they (1) were enrolled as members in one of the three participating healthcare systems: HealthPartners Institute (HP), Kaiser Permanente Georgia (KPGA), or Kaiser Permanente Mid-Atlantic States (KPMAS), (2) were diagnosed with, or had a positive lab value for either influenza-like illness (ILI) or COVID-19, or (3) reported 2 + viral respiratory symptoms (cough, migraine, shortness of breath, fever, myalgia, or fatigue) captured during their medical visit. Patients were excluded if they were hospitalized or ventilated at the time of diagnosis or positive lab value, were diagnosed with dementia, or if they had actively opted out of research at their respective healthcare systems. Electronic informed consent was obtained for all participants prior to the participants being able to access the FLU-PRO survey. All methods and protocols were carried out in accordance with human subjects research guidelines. Protocols were approved by the HealthPartners Institute, Kaiser Permanente Georgia, and Kaiser Permanente Mid-Atlantic States’ Institutional Review Boards.

### Recruitment

Each integrated healthcare system utilized their robust electronic medical record (EMR) to determine member eligibility based on the inclusion and exclusion criteria. Standardized eligibility criteria were disseminated in an EMR-based algorithm shared across sites. Recruitment occurred within 72-hours of meeting the eligibility criteria. The algorithm generated a daily report of eligible members. Rolling recruitment began in February 2021 and continued through May 15, 2021.

**Fig. 1:**
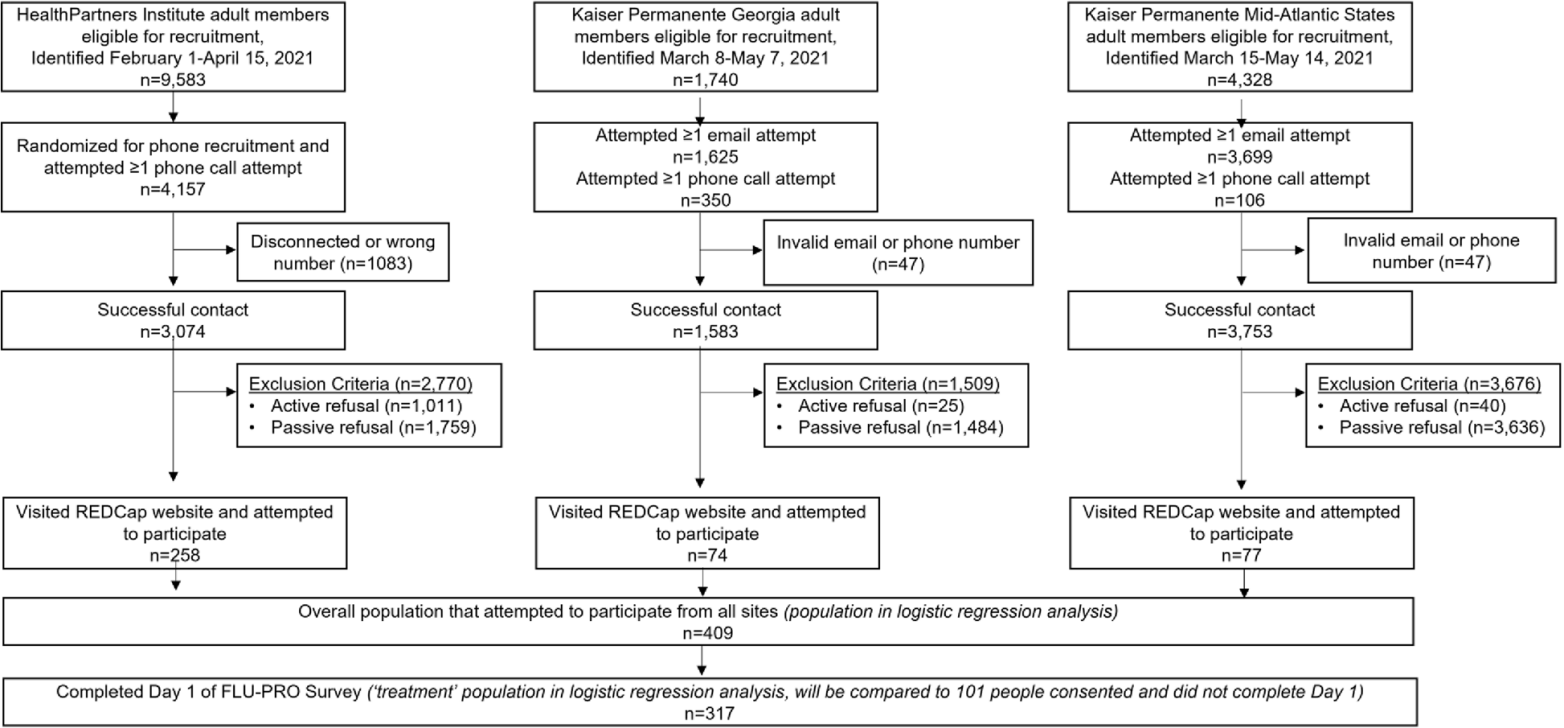
Flow diagram of participant eligibility, recruitment, consent, and day 1 survey completion

Recruitment methodology was optimized for each healthcare system’s member population. HP conducted phone recruitment on a randomized list of eligible members. HP relied only on phone recruitment which occurred Monday-Friday from February 1, 2021, through April 15, 2021. KPGA and KPMAS used a hybrid of email and phone recruitment for daily, Monday-Friday recruitment outreach. All eligible members with a registered email address received an initial recruitment email. Targeted phone calls were then completed based on the following prioritization: (1) no listed email address, (2) ILI lab positive or diagnosis, and (3) COVID-19 lab positive or diagnosis. KPGA began recruitment on March 8, 2021, and KPMAS began on March 15, 2021. Each Kaiser Permanente site completed 9-weeks of recruitment. All interested participants were directed to the online survey to provide online consent and begin completing the FLU-PRO Plus 14-day survey questionnaires. Figure 1 shows the site-specific flow diagram of participant recruitment, consent, and FLU-PRO Plus Day 1 completion.

### Data collection

FLU-PRO Plus is an electronic PROM developed to assess symptoms related to ILI and COVID-19. The survey is designed to be completed daily by the participant for 14-days and asks the participant to rate the frequency and intensity of 34 symptoms, in the past 24-hours using a five-point Likert scale [28, 36]. The FLU-PRO Plus survey was administered using the HIPAA compliant electronic data capture software REDCap hosted by HP. After eligible adults were recruited and received the link to the REDCap-based FLU-PRO Plus, the participants were instructed to complete electronic, informed consent and were subsequently asked if they were still experiencing symptoms. We ascertained basic patient characteristics from each integrated healthcare system’s administrative databases for all eligible adults to describe the demographic differences between adults that were eligible, were successfully contacted, completed consent, and completed FLU-PRO Plus Day 1.

Our outcomes were focused on describing the eligible population that were (1) successfully contacted and (2) completed Day 1 FLU-PRO Plus. Eligible adults were considered to have a successful contact (yes/no) if the telephone number on file was still working or an email was sent to the individual’s registered email address and an error message did not occur. Completion of FLU-PRO Plus Day 1 was defined as ‘yes’ or ‘no’ and was determined if the participant used the FLU-PRO Plus link to log in after completing consent. We examined the odds of being successfully contacted and the odds of completing FLU-PRO Plus Day 1 among all eligible adults. Among the successfully contacted individuals, we examined the odds of completing FLU-PRO Plus Day 1.

Patient characteristics were collected from the EMR and were defined at the time of eligibility. Age at the time of eligibility was classified as a mean (SD) and categorized as 18–34, 35–64, and ≥ 65 years. Participant race from the EMR was categorized as ‘Asian’, ‘Black or African American’, ‘White’, ‘Other’, or ‘Unknown race’. Other race was defined as a race outside of the named categories and for participants reporting multiple races. ‘Unknown race’ was assigned if a participant did not have a reported race in the EMR. Gender (‘male’ vs. ‘female’) and Hispanic (‘yes’ vs. ‘no’) ethnicity were self-reported in the electronic medical record. Recruitment mode was captured at each site at the time of recruitment. Diagnosis, initially defined at the time of enrollment, was confirmed and validated by lab records 30-days after initial diagnosis record and was defined as either COVID-19 or influenza-like illness positive.

### Statistical analysis

Descriptive statistics were calculated to describe the patient characteristics for participants that were eligible for FLU-PRO Plus, successfully contacted, had attempted to log in to the FLU-PRO Plus website, and completed FLU-PRO Plus Day 1 across all three sites. We assessed the FLU-PRO Plus survey days by patient characteristics overall and the total of survey days completed across each site. Bivariable and multivariable logistic regression investigated the patient characteristics associated with (1) successful contact and (2) FLU-PRO Plus Day 1 completion. PROC GLIMMIXX accounted for site-level clustering and treated each healthcare system as a random effect in the logistic regression models. All analyses were performed using SAS 9.4 ©.

## Results

We identified a total of 15,650 eligible participants during the enrollment period: 9,582 from HP, 1,740 from KPGA, and 4,328 from KPMAS. HP randomized half of their eligible population (4,157 adults) for phone recruitment and successfully contacted 3,074. A total of 258 eligible adults from HP visited the REDCap website and attempted to participate in FLU-PRO Plus. KPGA successfully contacted 1,583 participants, of whom 74 eligible adults visited the REDCap website and attempted to participate in FLU-PRO Plus. KPMAS successfully contacted 3,753 participants and had 77 eligible adults visit the REDCap website and attempt to participate in FLU-PRO Plus. Among the total of 409 eligible adults who attempted to participate in FLU-PRO Plus, 317 completed FLU-PRO Plus Day 1.

Descriptive statistics reported the patient characteristics by site that were (1) eligible to participate in FLU-PRO Plus, (2) successfully contacted, (3) visited the website, and attempted to participate in FLU-PRO Plus, and (4) completed FLU-PRO Plus Day 1 (Table 1). Adults meeting the inclusion criteria and eligible to participate in FLU-PRO Plus were aged 35–64 years old (HP = 54.4%, KPGA = 62.9%, KPMAS = 56.3%); similar to the age distribution of the participants completing FLU-PRO Plus Day 1 (HP = 67.0%, KPGA = 78.0%, KPMAS = 51.6%). A higher percentage of females completed FLU-PRO Plus Day 1 across all three sites (HP = 66.5%, KPGA = 76.0%, KPMAS = 73.4%). HP had a higher number of White adults who were eligible (*n* = 7,443; 77.7%), successfully contacted (*n* = 2,507; 81.6%), visited the website and attempted to participate (*n* = 219; 84.9%), and completed FLU-PRO Plus Day 1 (*n* = 178; 87.7%), compared to other race/ethnicity groups. Both KPGA and KPMAS had a higher percentage of Black and African American adults that were successfully contacted (KPGA = 50.7%, KPMAS = 51.6%) compared to other race groups. A higher percentage of Black and African American KPGA adults visited the website and attempted to participate (52.7%) and completed FLU-PRO Plus Day 1 (54.0%) compared to other KPGA race groups.

**Table 1:** Descriptive statistics of patient characteristics at time of eligibility across three integrated healthcare systems, February-May 2021

HealthPartners Institute				Kaiser Permanente Georgia				Kaiser Permanente Mid-Atlantic States		
FLU-PRO Plus Eligible Population (*n* = 9582)	Successfully contacted (*n* = 3074)	Visited Website to Login (*n* = 258)	Completed Day 1 (*n* = 203)	FLU-PRO Plus Eligible Population (*n* = 1740)	Successfully contacted (*n* = 1583)	Visited Website to Login (*n* = 74)	Completed Day 1 (*n* = 50)	Successfully contacted (*n* = 3753)	Visited Website to Login (*n* = 77)	Completed Day 1 (*n* = 64)
Patient Characteristics at time of eligibility n (%)										
48.2 (17.9)	51.2 (17.7)	51.7 (14.7)	51.8 (14.4)	47.2 (16.1)	46.9 (51.7)	47.5 (13.6)	47.8 (11.9)	43.6 (15.6)	39.3 (14.9)	39.2 (14.4)
2542 (26.5)	643 (20.9)	38 (14.7)	27 (13.3)	417 (24.0)	383 (24.2)	14 (18.9)	8 (16.0)	1270 (33.8)	35 (45.5)	29 (45.3)
5211 (54.4)	1702 (55.4)	171 (66.3)	136 (67.0)	1094 (62.9)	1002 (63.3)	52 (70.3)	39 (78.0)	2117 (56.4)	39 (50.7)	33 (51.6)
1829 (19.1)	729 (23.7)	49 (19.0)	40 (19.7)	229 (13.2)	198 (12.5)	8 (10.8)	3 (6.0)	366 (9.8)	3 (3.9)	2 (3.1)
5497 (57.4)	1777 (57.8)	165 (64.0)	135 (66.5)	1085 (62.4)	994 (62.8)	50 (67.6)	38 (76.0)	2172 (57.9)	58 (75.3)	47 (73.4)
4085 (42.6)	1297 (42.2)	93 (36.1)	68 (33.5)	655 (37.6)	589 (37.2)	24 (32.4)	12 (24.0)	1581 (42.1)	19 (24.7)	17 (26.6)
349 (3.6)	97 (3.2)	5 (1.9)	1 (0.5)	66 (3.8)	59 (3.7)	1 (1.4)	1 (2.0)	461 (12.3)	9 (11.7)	7 (10.9)
984 (10.3)	269 (8.8)	20 (7.8)	15 (7.4)	890 (51.2)	803 (50.7)	39 (52.7)	27 (54.0)	1937 (51.6)	26 (33.8)	17 (26.6)
529 (5.5)	134 (4.4)	10 (3.9)	6 (3.0)	15 (0.9)	14 (0.9)	1 (1.4)	1 (2.0)	74 (2.0)	4 (5.2)	4 (6.3)
277 (2.9)	67 (2.2)	4 (1.6)	3 (1.5)	252 (14.5)	222 (14.0)	8 (10.8)	6 (12.0)	343 (9.1)	3 (3.9)	3 (4.7)
7443 (77.7)	2507 (81.6)	219 (84.9)	178 (87.7)	517 (29.7)	485 (30.6)	25 (33.8)	15 (30.0)	938 (25.0)	35 (45.5)	33 (51.6)
323 (3.4)	73 (2.4)	10 (3.9)	7 (3.5)	86 (4.9)	80 (5.1)	3 (4.1)	2 (4.0)	381 (10.2)	5 (6.5)	4 (6.3)
9456 (98.7)	3036 (98.8)	257 (99.6)	203 (100.0)	1672 (96.1)	1525 (96.3)	71 (96.0)	48 (96.0)	3753 (100.0)	77 (100.0)	64 (100.0)
4157 (43.4)	3074 (100.0)	258 (100.0)	203 (100.0)	5 (0.3)	0 (0.0)	0 (0.0)	0 (0.0)	104 (2.8)	0 (0.0)	0 (0.0)
N/A	N/A	N/A	N/A	1280 (73.6)	1269 (80.2)	45 (60.8)	33 (66.0)	3649 (97.2)	77 (100.0)	64 (100.0)
N/A	N/A	N/A	N/A	345 (19.8)	314 (19.8)	29 (39.2)	17 (34.0)	0 (0.0)	0 (0.0)	0 (0.0)
5425 (56.6)	N/A	N/A	N/A	110 (6.3)	N/A	N/A	N/A	N/A	N/A	N/A
4317 (45.1)	1196 (38.9)	136 (52.7)	104 (51.2)	1308 (75.2)	1202 (75.9)	61 (82.4)	42 (84.0)	3506 (93.4)	72 (93.5)	59 (92.2)
5265 (55.0)	1878 (61.1)	122 (47.3)	99 (48.8)	432 (24.8	381 (24.1)	13 (17.6)	8 (16.0)	247 (6.6)	5 (6.5)	5 (7.8)

**Fig. 2:**
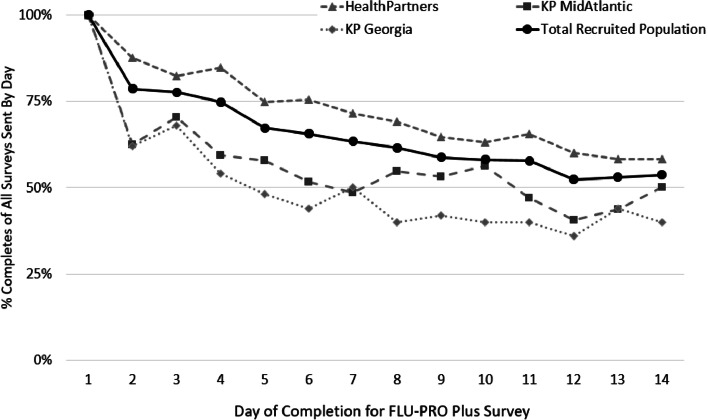
Daily progress of FLU-PRO Plus survey completion among all individuals who consented and completed Day 1, over the course of the 14-day survey period, overall and stratified by site

Table 2 reports the frequency of adults that visited the website and attempted to participate, and completed FLU-PRO Plus Day 1, 3, 7, 10, and 14. There were 118 adults from HP that completed FLU-PRO Plus Day 14 compared to 20 and 32 adults from KPGA and KPMAS, respectively. Females and adults aged 34–65 years mostly completed FLU-PRO Plus Day 1 through 14. There were 122 (29.8%) participants that were recruited through email only and visited the FLU-PRO Plus website; 97 of those individuals completed FLU-PRO Plus Day 1. Among the 317 individuals that completed FLU-PRO Plus Day 1, 205 (67.5%) were diagnosed with COVID-19; 112 adults diagnosed with COVID-19 completed FLU-PRO Plus Day 14. Among all individuals completing FLU-PRO Plus Day 1, there was a gradual decline of percent completion of FLU-PRO Plus surveys throughout the 14-day survey period (Fig. 2).

**Table 2:** Descriptive statistics on the FLU-PRO Plus steps completed

	Attempted Website Login (*n* = 409)	Day 1 FLU-PRO Plus *n* = 317 (77.5%)	Day 3 FLU-PRO Plus *n* = 246 (77.6%)^a^	Day 7 FLU-PRO Plus *n* = 201 (63.4%)^a^	Day 10 FLU-PRO Plus *n* = 184 (58.0%)^a^	Day 14 FLU-PRO Plus *n* = 170 (53.6%)^a^
**INTEGRATED HC SYSTEM**						
HealthPartners Institute	258 (63.1)	203 (64.0)	167 (67.9)	145 (72.1)	128 (69.6)	118 (69.4)
Kaiser Permanente Georgia	74 (18.1)	50 (15.8)	34 (13.8)	25 (12.4)	20 (10.9)	20 (11.8)
Kaiser Permanente Mid-Atlantic States	77 (18.8)	64 (20.2)	45 (18.3)	31 (15.4)	36 (19.6)	32 (18.8)
Age at time of eligibility, mean (SD)	48.6 (15.2)	48.6 (14.9)	49.7 (15.2)	51.3 (14.6)	51.7 (13.9)	52.8 (13.8)
**Age CATEGORIES**						
18–34	87 (21.3)	64 (20.2)	48 (19.5)	29 (14.4)	22 (12.0)	20 (11.8)
35–64	262 (64.1)	208 (65.6)	158 (64.2)	136 (67.6)	129 (70.1)	118 (69.4)
≥ 65	60 (14.7)	45 (14.2)	40 (16.3)	36 (17.9)	33 (17.9)	32 (18.8)
**GENDER**						
Female	273 (66.8)	220 (69.4)	165 (67.1)	135 (67.2)	123 (66.9)	112 (65.9)
Male	136 (33.3)	97 (30.6)	81 (32.9)	66 (32.8)	61 (33.2)	58 (34.1)
**RACE**						
Asian	15 (3.7)	9 (2.8)	4 (1.6)	3 (1.5)	1 (0.5)	2 (1.2)
Black/African American	85 (20.8)	59 (19.6)	29 (11.8)	29 (14.4)	25 (13.6)	24 (14.1)
Other	15 (3.7)	11 (3.5)	10 (4.1)	6 (3.0)	5 (2.7)	1 (0.6)
Unknown	15 (3.7)	12 (3.8)	11 (4.5)	5 (2.5)	3 (1.6)	5 (2.9)
White	279 (68.2)	226 (71.3)	192 (78.1)	158 (78.6)	150 (81.5)	138 (81.2)
Hispanic	18 (4.4)	13 (4.1)	10 (4.1)	7 (3.5)	5 (2.7)	3 (1.8)
Primary Language, English	405 (99.0)	315 (99.4)	244 (99.2)	200 (99.5)	184 (100.0)	169 (99.4)
**RECRUITMENT MODE**						
Phone	258 (63.1)	203 (64.0)	167 (67.9)	145 (72.1)	128 (69.6)	118 (69.4)
Email	122 (29.8)	97 (30.6)	69 (28.1)	50 (24.9)	49 (26.6)	47 (27.7)
Email + Phone	29 (7.1)	17 (5.4)	10 (4.1)	6 (3.0)	7 (3.8)	5 (2.9)
**DIAGNOSIS**						
COVID-19	269 (65.8)	205 (64.7)	155 (63.0)	126 (62.7)	119 (64.7)	112 (65.9)
Influenza-like illness (ILI)	140 (34.2)	112 (35.3)	91 (37.0)	75 (37.3)	65 (35.3)	58 (34.1)

^a^Percentages were calculated using the number of participants that completed the specified day of FLU-PRO Plus survey (numerator), divided by the number of participants that completed FLU-PRO Plus Day 1 (denominator).

Multivariable logistic regression evaluated the patient characteristics associated with adults being successfully contacted and completing FLU-PRO Plus Day 1. Table 3 reports that among those eligible for FLU-PRO Plus, adults aged 35–64 (OR = 1.15, 95% CI 1.05, 1.26) and aged ≥ 65 (OR = 1.15, 95% CI 1.02, 1.30) had higher odds of being successfully contacted compared to adults aged 18–34. Black and African American adults (OR = 0.74, 95% CI 0.66, 0.83) and adults diagnosed with COVID-19 (OR = 0.73, 95% CI 0.68, 0.80) had lower odds of being successfully contacted compared to their counterparts. Among those eligible for FLU-PRO Plus, adults aged 35–64 (OR = 1.59, 95% CI 1.20, 2.11), females (OR = 1.76, 95% CI 1.38, 2.25), and adults diagnosed with COVID-19 (OR = 1.40, 95% CI 1.09, 1.80) had higher odds of completing FLU-PRO Plus Day 1; Asian adults (OR = 0.36, 95% CI 0.18, 0.72) and Black and African American adults (OR = 0.46, 95% CI 0.33, 0.64) had lower odds compared to White adults. Similarly, among adults successfully contacted, adults aged 35–64 (OR = 1.40, 95% CI 1.05, 1.87), females (OR = 1.77, 95% CI 1.38, 2.27), and adults diagnosed with COVID-19 (OR = 1.66, 95% CI 1.27, 2.17) had higher odds of completing FLU-PRO Plus Day 1; Asian adults (OR = 0.38, 95% CI 0.19, 0.76) and Black and African American adults (OR = 0.33, 95% CI 0.19, 0.76) had lower odds completing FLU-PRO Pus Day 1, compared to White adults.

**Table 3:** Multivariable logistic regression^a^ investigating the odds of completing FLU-PRO Plus Day 1 among those who completed online consent

	Among everyone eligible				Among successfully contact	
Patient characteristics at time of eligibility	Odds of successfully contacted		Odds of Day 1 Flu-Pro response		Odds of Day 1 Flu-Pro response	
	OR	95% CI	OR	95% CI	OR	95% CI
**Age**						
18–34	Reference		Reference		Reference	
35–64	1.15	1.05, 1.26	1.59	1.20, 2.11	1.40	1.05, 1.87
≥ 65	1.15	1.02, 1.30	1.09	0.74, 1.61	0.87	0.58, 1.30
**Gender**						
Male	Reference		ref		ref	
Female	1.02	0.94, 1.10	1.76	1.38, 2.25	1.77	1.38, 2.27
**Race**						
Asian	0.85	0.71,1.02	0.36	0.18, 0.72	0.38	0.19, 0.76
Black/African American	0.74	0.66, 0.83	0.46	0.33, 0.64	0.46	0.33, 0.65
White	Reference		Reference		Reference	
Other	0.82	0.66, 1.01	0.73	0.37, 1.42	0.87	0.44, 1.72
Unknown	0.67	0.53, 0.81	0.44	0.22, 0.87	0.46	0.23, 0.92
**Hispanic ethnicity** (reference = no)	0.75	0.60, 0.94	1.09	0.46, 1.82	0.99	0.49, 2.01
**Diagnosis**						
COVID-19	0.73	0.68, 0.80	1.40	1.09, 1.80	1.66	1.27, 2.17
Influenza-like illness (ILI)	Reference		Reference		Reference	

^a^Logistic regression models were analyzed on the total population referenced in each column. The multivariable logistric regression models all controlled for age, gender, patient-reported race, Hispanic ethnicity, and viral respiratory diagnosis.

## Discussion

Our multi-site feasibility study aimed to evaluate the feasibility of routine surveillance of respiratory viral syndromes in clinical practice and attempted to contact 15,650 eligible adults across three integrated healthcare systems. Of the eligible adults successfully contacted, we had 3.7% complete FLU-PRO Plus Day 1. Among those who completed Day 1 of FLU-PRO Plus, 77.6% completed Day 7 and 53.6% completed Day 14 of the survey. Compared to their eligible counterparts that were successfully contacted, females and people aged 35–60 years were more likely to complete Day 1 of FLU-PRO Plus.

PROMs play an important role in patient-centered care that can lead to patient goal setting, improvement in patient engagement, improve effectiveness of patient-provider interaction, and increased patient self-efficacy [2, 19, 26, 32, 37–39]. However, despite their utility and benefits to both the patient and healthcare system, PROM efficacy and implementation in patient care has been mostly focused within oncology [2]. The web-based Symptom Tracking and Reporting ePROM system (STAR) tracked chemotherapy patients’ symptoms on a weekly basis and was shown to significantly reduce the number of emergency department visits and in-patient hospitalizations, improve health-related quality of life, and significantly improve overall survival in the group of patients randomized to use the PROM [9, 10, 14]. Increased odds of survival were also seen in the e-Follow-Up application PROM for adults undergoing lung cancer treatment [9–12]. FLU-PRO Plus is a reliable and valid PROM that provides standardized questions on seven symptom domains that could be used across patient populations. FLU-PRO Plus has also been translated in to > 23 languages including Spanish, Nepalese, Hindi, Zulu, Portuguese, Korean, French, Russian, and Vietnamese. The utilization of FLU-PRO Plus globally would provide healthcare providers and scientists the ability to complete more robust disease and symptom surveillance across populations, locally and internationally. A standardized mechanism of collecting symptom data would also enable more efficient tracking of virus mutations overtime, like we have seen with COVID-19 mutations and different strains increasing the odds of different symptoms [40].

PROMs may enable providers to more accurately differentiate between viral respiratory symptoms and track seasonal illnesses [31, 36]. The onset of the SARS-CoV-2 (COVID-19) pandemic highlighted the importance of standardized, systematic, and comprehensive measures that accurately capture patient-reported viral respiratory symptoms [33]. Healthcare provider-developed questions may miss important information about respiratory infections or inaccurately represent the frequency of duration of reported symptoms [33–35, 41]. FLU-PRO is a PROM that was developed and validated in patients with acute, laboratory-confirmed influenza and influenza like illness [28, 42]. FLU-PRO scores were reliable, reproducible, and reported high internal consistencies across the six original domains (nose, throat, eyes, chest/respiratory, gastrointestinal, body/systemic) [21]. The development of FLU-PRO Plus occurred at the onset of the COVID-19 pandemic and included a seventh domain of senses (loss of taste and smell) in May 2020 [36]. The authors enrolled COVID-19 positive patients from March 2020 through June 2021 that completed Day 1 of either FLU-PRO (March 2020-April 2020) or FLU-PRO Plus (May 2020-June 2021) [36]. The Cronbach’s alpha reliability across the seven FLU-PRO Plus’s domains remained above 0.86 and the responsiveness to the 14-days of survey showed a steady decrease across time [36], similar to the responsiveness our feasibility study showed.

PROMs have been shown to improve patient survival, quality of life, and symptom management [9, 12, 14, 27]. Healthcare systems also benefit from the use of PROM through more efficient use of limited healthcare resources, reduction of patient-reimbursement for travel, fewer emergency department visits, and improved treatment adherence [2, 26]. FLU-PRO Plus could provide a healthcare system the ability to monitor their patients’ ongoing symptoms remotely, leading to better patient care, improved allocation of healthcare resources, and reduced risk of disease transmission during viral respiratory disease outbreaks. In addition to providing better individual-level patient care, healthcare systems could pair the FLU-PRO Plus PROM with their robust EMR administrative database to aggregate symptom tracking across patients. Aggregating patient-reported viral respiratory symptoms across patients, would enable the healthcare system to track any viral respiratory infections, such as the influenza, rhinovirus, or COVID-19 across their patient population, overall, and geographically stratified to better assess local outbreaks and properly allocate resources to clinics in the most need. Integrating FLU-PRO Plus within a healthcare system would allow the healthcare system to compare and improve provider-level care and track the patient’s quality of life changes over time.

The COVID-19 pandemic facilitated a renewed interest among healthcare systems, patients, and policy makers to focus on telemedicine that may be sustained beyond the pandemic. U.S. policy makers have begun to address improving access to affordable, reliable, and high-speed internet to every American [43] as more healthcare systems implement telemedicine practices [24, 44–46]. As the use of telemedicine and eHealth interventions continue to increase [47], FLU-PRO Plus and other PROMS that are designed to ask lay questions in a short survey format may be the best option to provide remote surveillance while accounting for possible eHealth literacy disparities [48, 49]. The FLU-PRO Plus feasibility study, across three diverse sites, was able to show the proportion of people eligible, successfully contacted, and completed Day 1 remained constant across patient demographics except for age ≥ 65 that had a lower Day 1 completion compared to other ages. Integration of FLU-PRO Plus within the clinical workflow could potentially reduce the ‘digital divide’ or eHealth Literacy disparity by providing patients the opportunity to be trained to use and answer the prompts during their in-clinic, Day 1 visit. A follow-up study should focus on the implementation mechanisms that would enhance the uptake of FLU-PRO Plus, capture the utility across subpopulations, and gather feedback from patient- and clinical-stakeholders on the real-world use of FLU-PRO Plus.

While the results demonstrate the feasibility of using FLU-PRO Plus across three integrated healthcare systems, the study has notable limitations. First, because our recruitment occurred between February through May 2021, our sample does not account for the recent COVID-19 variants and has limited sample size for patients diagnosed with influenza or influenza like illnesses. Second, the generalizability of the findings is limited due to the small sample sizes, recruitment restrictions, short sampling timeframe, and limited geographic reach. In this feasibility study, each of the three integrated healthcare systems completed recruitment based on institutional guidelines and resources. While we were able to test different recruitment methodologies and determine that combined email and phone recruitment may be the most successful strategy, it made defining the recruitment rate challenging. Future studies should apply the lessons learned from this feasibility study and design a robust email and phone recruitment strategy. Future studies should also include a prolonged recruitment period to increase the recruitment size and diversity across viral respiratory diseases and variants. Third, our analysis was limited to Day 1 of FLU-PRO Plus completion because we noticed participants were no longer completing the daily survey once their symptoms subsided and they returned to baseline health. Therefore, we decided an analysis on Day 14 completion would not be accurate and a revision of FLU-PRO Plus should include an opening question asking the individual to report if their symptoms have subsided to accurately capture the information on symptom duration. Fourth, our study excluded patients hospitalized at the time of eligibility and limits our findings to patients with less severe viral respiratory infections, which comprised most of the viral respiratory infections in 2021 [50–52]. Fifth, our study was limited on the socioeconomic variables collected at the participant-level and could not fully assess if a digital or eHealth literacy disparities were present among participants. Future studies should focus on collecting more granular socioeconomic and neighborhood-level variables, in addition to e-skills capability, to improve utilization across population groups. Sixth, this assessment did not differentiate symptom patterns between viral respiratory infections such as COVID-19 and influenza-like illnesses. Additional studies are needed to evaluate if symptom scores could capture distinct patterns in intensity and duration across viral respiratory illnesses.

## Conclusion

Patient-reported outcome measures can improve patient care and quality of life and reduce the strain of limited healthcare systems resources. FLU-PRO Plus is a PROM focused on tracking viral respiratory illnesses including influenza, respiratory syncytial virus, rhinovirus, enterovirus, and coronaviruses. Our study reports on the feasibility of patients across three integrated healthcare systems utilizing FLU-PRO Plus to monitor their respiratory symptoms for 14-days and the patient characteristics associated with completing FLU-PRO Plus Day 1. Future FLU-PRO Plus studies should develop an implementation strategy to fully integrate FLU-PRO Plus within clinical care and patient management. As the acceptability and utilization of telemedicine and smart devices continue to increase, healthcare systems should strongly consider broad implementation of PROMs to collect patient-reported information that may lead to improved patient care and resource allocation.

## Supplementary Information

**Additional file 1:** **Table 1.** Bivariablelogistic regression investigating the odds of completing FLU-PRO Plus Day 1among those who completed online consent.

## Data Availability

The data underlying this article cannot be shared publicly due to the privacy of individuals and integrated healthcare system members that participated in the study. The derived data will be shared on reasonable request to the corresponding author.

## Abbreviations

HP: HealthPartners Institute
ILI: Influenza-like-illness
KPGA: Kaiser Permanente Georgia
KPMAS: Kaiser Permanente Mid-Atlantic States
PROM: Patient-reported outcome measures
