# Correction: Systematic surveillance of patient-reported symptoms of viral respiratory tract infectious Syndromes in diverse populations

**DOI:** 10.1186/s12913-023-09096-1

**Published:** 2023-01-26

**Authors:** Jennifer C. Gander, Ella Chrenka, Lee Cromwell, Anjali R. Truitt, Musu Sesay, Marni Segall, Sandra A. Amouzou, Alexander F. Hudgins, Prasanthi Kodthala, Douglas Roblin, Adrienne N. Deneal, Thomas Whiting, John H. Powers, Brian C. Martinson

**Affiliations:** 1 Center for Research and Evaluation, Kaiser Permanente Georgia, Atlanta, GA USA; 2 HealthPartners Institute, Bloomington, MN USA; 3 Mid-Atlantic Permanente Research Institute, Kaiser Permanente Mid-Atlantic States, Rockville, MD USA; 4 George Washington University School of Medicine, Washington, DC USA

**Correction: BMC Health Services Research 22, 1591 (2022)**

**https://doi.org/10.1186/s12913-022-08991-3**

Following publication of the original article [1], the authors identified an error in the author name (middle initial) of John H. Powers.

The incorrect author name is: John D. Powers

The correct author name is: John H. Powers

In addition, the authors identified an error in the affiliation of John H. Powers (affiliation 4): it was corrected from ‘Clinical Research Directorate, Frederick National Laboratory for Cancer Research’ to ‘George Washington University School of Medicine’.

The author group and list of affiliations has been updated above and the original article [1] has been corrected.
