# Supplementary material for: Systematic surveillance of patient-reported symptoms of viral respiratory tract infectious Syndromes in diverse populations
Source: BMC Health Serv Res. 2022 Dec 29;22:1591. doi: 10.1186/s12913-022-08991-3 (PMC9797889; doi:10.1186/s12913-022-08991-3)
Supplement: Supplementary file 1 — Additional file 1: Table 1. Bivariablelogistic regression investigating the odds of completing FLU-PRO Plus Day 1among those who completed online consent. [file 12913_2022_8991_MOESM1_ESM.docx]

Supplemental Table 1. Bivariable logistic regression investigating the odds of completing FLU-PRO Plus Day 1 among those who completed online consent.

|  | **Among everyone eligible** | | | | | **Among successfully contact** | |
| --- | --- | --- | --- | --- | --- | --- | --- |
| **Patient characteristics at time of eligibility** | **Odds of successfully contacted** | | **Odds of Day 1 Flu-Pro response** | | | **Odds of Day 1 Flu-Pro response** | |
|  | **OR** | **95% CI** | **OR** | **95% CI** | **OR** | | **95% CI** |
| Age |  |  |  |  |  | |  |
| 18-34 | Reference | | Reference | | Reference | | |
| 35-64 | 1.18 | 1.08, 1.29 | 1.61 | 1.21, 2.13 | 1.40 | | 1.05, 1.87 |
| ≥65 | 1.31 | 1.17, 1.47 | 1.16 | 0.79, 1.71 | 0.88 | | 0.59, 1.31 |
| Gender |  |  |  |  |  | |  |
| Male | Reference | | Reference | | Reference | | |
| Female | 1.03 | 0.95, 1.11 | 1.65 | 1.29, 2.10 | 1.64 | | 1.29, 2.10 |
| Race |  |  |  |  |  | |  |
| Asian | 0.84 | 0.70, 1.01 | 0.37 | 0.19, 0.73 | 0.39 | | 0.19, 0.77 |
| Black/African-American | 0.71 | 0.64, 0.80 | 0.50 | 0.36, 0.69 | 0.52 | | 0.37, 0.73 |
| White | Reference | | Reference | | Reference | | |
| Other | 0.69 | 0.57, 0.84 | 0.71 | 0.39, 1.31 | 0.90 | | 0.48, 1.67 |
| Unknown | 0.56 | 0.46, 0.67 | 0.43 | 0.24, 0.79 | 0.47 | | 0.23, 0.87 |
| **Hispanic ethnicity** (reference=no) |  |  |  |  |  | |  |
| **Diagnosis** |  |  |  |  |  | |  |
| COVID-19 | 0.73 | 0.68, 0.80 | 1.28 | 0.99, 1.65 | 1.61 | | 1.24, 2.09 |
| Influenza-like illness (ILI) | Reference | | Reference | | Reference | | |
| ^a^Logistic regression models were analyzed on the total population referenced in each column. The bivariate logistric regression models controlled for age, gender, patient-reported race, Hispanic ethnicity, and viral respiratory diagnosis seperately. | | | | | | | |
